# Transforming dementia research into practice: a multiple case study of academic research utilization strategies in Dutch Alzheimer Centres

**DOI:** 10.1186/s12961-024-01266-9

**Published:** 2025-01-06

**Authors:** Eden Meng Zhu, Martina Buljac-Samardžić, Kees Ahaus, Robbert Huijsman

**Affiliations:** Erasmus School of Health Policy & Management, PO Box 1738, 3000 DR Rotterdam, Netherlands

**Keywords:** Knowledge translation, Implementation science, Research impact, Dementia

## Abstract

**Background:**

Non-pharmacological dementia research products, such as social and behavioural interventions, are generated in traditional university settings. These often experience challenges to impact practices that they were developed for. The Netherlands established five specialized academic health science centres, referred to as Alzheimer Centres, to structurally coordinate and facilitate the utilization of dementia research knowledge. This study leverages implementation science to systematically explore the research utilization strategies used by academic researchers from each Alzheimer Centre, on the basis of the “knowledge-to-action” (KTA) framework that includes knowledge creation, adaptation, dissemination and implementation.

**Methods:**

Individual semi-structured qualitative interviews were conducted with 29 respondents across the five Alzheimer Centres in the Netherlands. Participants were selected through purposive (snowball) sampling. Interviews were conducted in-person and virtually through Microsoft Teams, and all were audio-recorded and transcribed verbatim. Data analysis was guided by the dimensions of the KTA framework.

**Result:**

There was a high variation in the strategies used across the five Alzheimer Centres to bring non-pharmacological dementia research into practice. Selected strategies in each Centre were influenced by the typology of research products produced and the Centres’ organizational heritage. The knowledge creation and adaptation phases were mainly facilitated by funders’ guidance towards research impact and research product co-creation with patients and implementing organizations. Dissemination and implementation phases were often facilitated through utilizing support from university-based technology transfer offices to facilitate implementation and valorization and establishing and strategically leveraging formal infrastructure, such as public–private partnerships and professional collaborative networks.

**Conclusions:**

Successful research utilization requires evolving researcher competencies to meet environmental demands and facilitating co-creation with research end-users and implementing partners. Understanding external determinants influencing research utilization in the Dutch dementia research ecosystem is crucial for capacity-building and aligning cross-sector agendas. The KTA framework appears to reveal the intricacies of research utilization, guiding future studies to explore strategies employed across various contexts.

**Supplementary Information:**

The online version contains supplementary material available at 10.1186/s12961-024-01266-9.

## Background

Non-pharmacological dementia research knowledge products, such as eHealth interventions, psycho-education programmes and diagnostic tools [1], are often siloed within academic settings and research domains. This contributes to the 17-year research-to-practice gap that delays research use and societal impact [2]. Knowledge and pragmatic tools that guide pharmacological drug discovery and development processes, including product production, validation, valuation and commercialization [3, 4]. These tools are considerably more mature compared with those available to guide researchers in non-pharmacological dementia research utilization [1, 5]. Formal research infrastructures, such as academic health science centres, have been developed as capacity-building initiatives to strengthen the research ecosystem and enhance research impact of such academic outputs [6–8]. The structure, governance and organizational dynamics within these formal research structures have been investigated [8], but previous studies have not structurally identified strategies, employed by academic researchers, to utilize research products [6, 7].

The concept of research utilization is also referred to as “knowledge translation”, “knowledge transfer”, or “knowledge mobilization” [9]. However, these terms are often inconsistently used in literature, and the explicit stages and strategies employed in the research utilization process are enigmatic in literature [10, 11]. Implementation science theories, models and frameworks help guide research utilization phases, identify specific strategies used and monitor and evaluate process outcomes [5, 12, 13]. The complex and iterative processes involved in delivering knowledge products to the intended end-users can be explored using process models, such as the knowledge-to-action (KTA) framework. This framework consists of two main components: the knowledge creation funnel and the knowledge action cycle [14]. It was originally developed by Graham et al. as a comprehensive “planned action model”, or “process model”, that guides the translation and transfer of academic research findings into real-world impact [9, 15, 16]. This framework was chosen to guide this study over other implementation process models, such as i-PARIHS [17], owing to its adaptability and wide application across various disciplines [14].

This study is guided by a four-phase research utilization model that includes knowledge creation, adaptation, dissemination, and implementation. These phases were derived on the basis of the two components of the KTA framework: the knowledge creation funnel and the knowledge action cycle [14]. The first component, the knowledge creation funnel, consists of three stages: knowledge inquiry, knowledge synthesis and the development of research knowledge tools and products, such as health education programmes and decision support tools [9]. The knowledge creation funnel guided the development of the first phase in this study’s research utilization model: ‘knowledge creation’. In this first phase, research output may be supported by a mode 1 approach, driven by funders and researchers, or a mode 2 approach, emphasizing society- and practice-focused research that encourages a participatory approach [7, 10]. A participatory approach uses input from various stakeholders to create equitable and feasible research knowledge products [18, 19]. Such stakeholders may include health practitioners in care settings and patients with lived experiences [18, 19].

The knowledge action cycle is the second component of the KTA framework. The seven iterative stages of this component are organized into three research utilization phases: “knowledge adaptation”, “knowledge dissemination”, and “knowledge implementation”. This is done to clearly explicate the strategies employed by researchers at each phase of the continuum. The phase of “knowledge adaptation” begins with a contextual needs assessment to determine environmental influences. This step is followed by knowledge adaptation activities to fit the research to the user context, such as co-designing and adapting research product components with local stakeholders and end-users [20–22]. For fundamental (biomedical) research products, this stage focuses on translating research findings into products, such as diagnostic tools, that are ready for implementation in clinics and other practice settings [23]. Knowledge adaptation should be delineated into intervention adaptation and implementation adaptation. Intervention adaptation refers to tailoring components of the intervention to fit the targeted user group. Implementation adaptation focuses on modifying the implementation plan to fit the contextual determinants (for example, available resources, organizational readiness) [24].

Knowledge dissemination is an implicit stage in the KTA trajectory, characterized as an activity of “end-of-grant knowledge translation” [9]. However, this study explicates dissemination as a critical stage used to translate knowledge to practice and policy [25]. Knowledge dissemination, the third phase in the research utilization model, is defined here as the transfer and exchange of knowledge beyond the boundaries of the research origin. This involves disseminating knowledge to the intended recipients, such as implementation agencies and patients [26]. Dissemination strategies can be categorized as a “push” or “pull” strategy, in which either (1) the knowledge producers proactively provide knowledge to their target recipients (for example, conducting training) or (2) the knowledge recipients seek knowledge to support their decision-making (for example, conducting a scoping review to inform policy) [27, 28]. Knowledge “exchange” strategies, also identified in “integrated knowledge translation” (IKT) literature, facilitate active co-creation and partnership engagement. These strategies focus on strengthening research infrastructure and health policies to effectively disseminate knowledge [29]. Notably, this stage is often facilitated prior to, or parallel to, the implementation process [12]. The last phase of the research utilization model is knowledge implementation. This consists of (1) assessing barriers and facilitators to implementation, (2) selecting and tailoring implementation strategies, (3) monitoring and evaluating implementation outcomes and (4) scaling and sustaining the intervention in the implementation setting [9].

A scoping review found 146 articles that mentioned the use of the KTA framework, but only 10 articles provided clear examples that demonstrated how the framework was used to guide implementation, all from the perspective of clinicians and healthcare practitioners [14, 16]. This study explores the perspective of researchers to structurally explore the activities performed to facilitate the research utilization process. This can inform the creation of theory-driven implementations strategies, which can explicate the knowledge utilization process to reduce implementation complexity and enhance process clarity [30]. In the Netherlands, five academic Alzheimer Centres were created to strengthen the dementia healthcare services and dementia research systems. These institutions also connect actors involved in research, treatment and education. Guided by the research utilization model and the KTA dimensions, this study aims to explore the unique research knowledge utilization activities of each Alzheimer Centre. It seeks to identify overarching strategies employed to create, adapt, disseminate and implement non-pharmacological dementia research to achieve research impact.

The main research questions include:

1. What strategies were used by the Alzheimer Centres to facilitate creation and adaptation of research findings into research products?
2. What strategies were employed to disseminate research products?
3. What strategies were used to facilitate the implementation of research products?

## Methods

### Study design

This study had a multiple case study design, guided by Yin [31], to explore the respective research knowledge utilization processes present in each Alzheimer Centre. This design was advantageous to identify “how” these processes occur and explore “why” certain strategies appeared in one context and were absent in others. To ensure reliability and validity, an in-depth analysis of each case was performed to identify the activities performed by each Alzheimer Centre at each stage of the KTA trajectory. Patterns in the activities were identified to inductively extrapolate research utilization strategies [32]. Cross-case comparisons (that is, cross-referencing) were used to compare strategies from each Alzheimer Centre and strengthen validity of findings across varied contexts. This approach also helped determine the contextual variables within each Alzheimer Centre that may influence the selection of certain strategies [31]. Results were developed on the basis of the findings from semi-structured interviews with key informants from each Alzheimer Centre.

### Setting

In the Netherlands, there are seven university medical centres (UMCs), located in Amsterdam, Rotterdam, Nijmegen, Groningen, Maastricht, Utrecht and Leiden, responsible for providing patient care, education and research [33]. Between 2000 and 2019, five UMCs have embedded Alzheimer Centres to centralize the creation of dementia research, education and care (diagnostic and treatment) and to provide tertiary support in each of their respective regional catchment areas [34, 35]. The Alzheimer Centres are academic health science centres and have a tripartite aim of providing patient care, education and research [8]. This structure promotes close multi-disciplinary collaboration and engagement between academic researchers and (clinical) practitioners [36].

Further, each Alzheimer Centre focuses on various areas and stages of dementia research, ranging from fundamental knowledge creation to applied research implementation and sustainment. These Alzheimer Centres were purposively selected as the focus of this study owing to their unique tripartite structure, their significant research output and their social and professional connectivity with other stakeholders within the Dutch dementia research ecosystem, detailed in Table 1.

**Table 1:** Description of Dutch dementia research ecosystem stakeholder groups

Stakeholders	Description of role and function in research ecosystem
ZonMw	ZorgOnderzoek Nederland (Care Research Netherlands; ZonMw) is a government-financed research funding agency that designs programmes that facilitate the allocation of public health research funding. In addition to providing funding, ZonMw performs activities including providing research impact planning guidance (for example, theory of change) and knowledge brokering between research teams, practice and policy
Dutch Organization for Scientific Research (NWO)	NWO is a government-financed research funding agency that ensures quality and innovation in science for a wider range of basic and interdisciplinary research areas
Alzheimer Nederland	Alzheimer Netherland is a charity and patient-representative organisation for people with dementia and their caregivers, as well as a dementia research funding agency and knowledge broker. Activities performed include advocating for better patient care, raising public awareness and facilitating informative support services, including: • “Dementia dialogues”: structured events that involve researchers and other stakeholders to discuss and share experiences, influence policies and strengthen support networks • “Alzheimer cafes”: informal community support meetings organized for people with dementia, caregivers and care and research professionals to share experiences, disseminate research and gather real-world perspectives
National knowledge institutions	National knowledge institutions (for example, Pharos and Vilans) enhance the research ecosystem by synthesizing evidence, guiding policy and ensuring knowledge translation to improve societal health outcomes. Researchers receive support from such institutes in knowledge brokering and translation
Health insurance agencies	Health insurers may support research utilization by financing, adopting and sustaining evidence-based practices. For example, *van thuis uit* is an initiative from CZ insurance that promotes ageing in place (home-based care)
Dutch Ministry of Health, Welfare and Sport (VWS)	VWS directs the national health research agenda and funds healthcare research, influences policy, sets standards and promotes innovations, significantly shaping healthcare quality and public health initiatives in the Netherlands. VWS established the National Dementia Strategy 2020–2030 to stimulate research (via ZonMw and NWO) aimed at improving quality of life for people with dementia and their caregivers. VWS also stimulates research through funding the *Stimuleringsregeling E-Health Thuis* (SET) initiative, which promotes the adoption and implementation of e-health solutions in home care settings
Professional associations or federations	Professional associations, including the Dutch Federation for Psychology and Dutch Federation for Neurologists, set professional standards, accredit educational programmes and impact research by promoting ethical guidelines and facilitating collaborations within their respective fields
	Verpleegkundigen and Verzorgenden Nederland (VVN) is a professional association for nurses, nursing assistants and professional carers in the Netherlands and support each group through professional standards, educational resources, advocacy, networking opportunities and promoting best practices. VVN supports occupational groups (for example, case managers) by providing professional development and guidance tailored to their needs within the healthcare industry

### Sample and recruitment

Research participants include programme managers and researchers employed by an Alzheimer Centre, specializing in a range of disciplines (for example, psychiatry, neuropsychology, epidemiology) that contribute to dementia research. The research team obtained permission from the leader(s) of each Alzheimer Centre, prior to the study, to conduct research in their organization. Individual participants were recruited using purposive and snowball sampling, identified through each Alzheimer Centre’s official website, official LinkedIn pages and through the referral of Alzheimer Centre leaders. These leaders also shared an introductory e-mail, on behalf of the research team, to inform and invite selected staff members to participate in this ongoing research project. Staff members responded with their intention to participate. Selected participants had a wide range of years of work experience and research area expertise, including developing fundamental research, social and behavioural programmes and digital health technologies. The variety of participant backgrounds included aimed to provide a representative sample of staff profiles and research portfolio of each Alzheimer Centre.

### Data collection

The research team, consisting of one PhD candidate and three university professors, conducted semi-structured qualitative interviews with five to six participants from each Alzheimer Centre. On average, each interview was 60 min and focused on the insights of one to two participants. The interview guide (see Additional file 1 Table 1) was developed with guidance from the stages of the KTA framework, focusing on the (1) mode of knowledge creation, (2) knowledge adaptation activities and (3) dissemination and implementation strategies. Each author listed in this study participated in developing the interview guide and conducting interviews. Topics and questions were pilot-tested in the first two interviews and remained the same. There were no repeated interviews needed. Informed consent forms were provided to each respondent prior to the interviews, detailing the scope of the project and the data management plan to provide transparency to participants. There were no withdrawals during the data collection process. All authors participated in conducting interviews. Interviews were conducted in-person or through video-conferencing between March 2023 and December 2023, and audio- and visual-recordings were made to ensure data accuracy during data transcription. Interviews were conducted until data saturation was reached (that is, responses became homogeneous and repetitive).

### Data analysis

Each interview recording was transcribed verbatim, and transcripts were sent to the respondents for final comments and approval. Sensitive information was redacted upon request. Each transcript was first examined individually, and repeated concepts were systematically labelled and thematically grouped to conduct content analysis using an abductive thematic coding approach on the basis of Timmermans and Tavory [37, 38]. First-order codes were deductively extracted and organized along the established dimensions of the KTA framework. Following, inductive thematic second-order codes were identified, extracted and analysed to explicate the research utilization strategies employed at each stage. This was the most appropriate approach given the dual research aim of identifying the novel strategies identified in this research context and their position in the KTA trajectory. The first author (E.M.Z.) conducted the initial first-order coding of the raw data. All authors were involved in developing and refining the second-order thematic codes to validate the final interpretation of themes. The final themes were used to develop research utilization strategies that informed the case description for each Alzheimer Centre. The coding framework can be found in Additional file 1 Table 2. The qualitative reporting in this study was guided by the COREQ checklist (see Additional file 1 Table 3) [39].

### Ethical approval

Ethical approval was obtained from the research ethics review committee at Erasmus University Rotterdam (ETH2223-0473), and all participants signed informed consent forms, detailing the scope of the study and the intended use of the data provided, to ensure research transparency and to protect the privacy rights of participants.

## Results

### Case descriptions

**Fig. 1:**
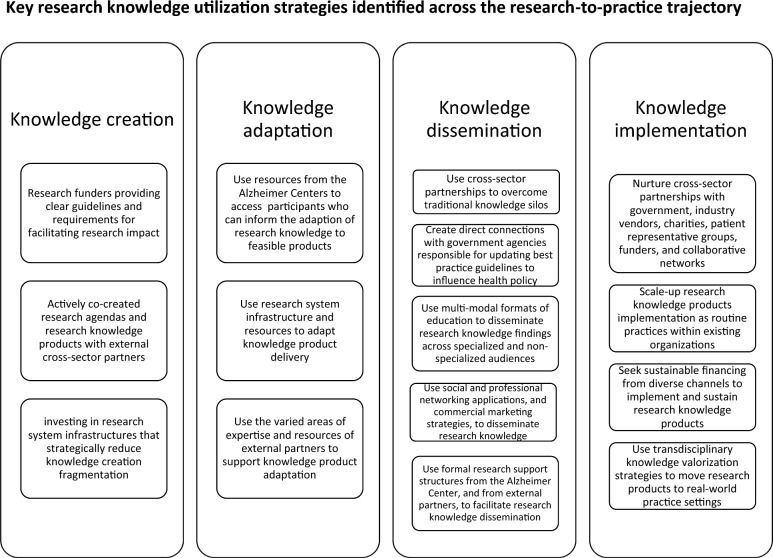
Key research knowledge utilization strategies identified across the research-to-practice trajectory

Data from 29 respondents were included in this study. The response rates for each Alzheimer Centre were Centre A: 5/6; Centre B: 6/8; Centre C: 6/9; Centre D: 6/10; and Centre E: 6/7. Respondents were early-career professionals (1–4 years of experience; 8/29; 27.6%), mid-career professionals (5–10 years of experience; 6/29; 20.7%) and senior-career professionals (10 + years of experience; 15/29; 51.7%). Each Centre facilitated collaboration between various UMC departments involved in dementia research, such as neurology, psychiatry, epidemiology, radiology and nuclear medicine and geriatrics. Each Centre invested in different research priority areas, including risk and prevention, etiology of dementia and dementia care services. Case descriptions for each Alzheimer Centre are presented in Table 2. Details on research utilization strategies utilized in each Alzheimer Centre are presented in Fig. 1 and Tables 3, 4, 5, 6, which serve as summary for the findings in each subsection of the Results.

**Table 2:** Alzheimer Centre case descriptions

*Centre A* Centre A was established in 2013 and serves a catchment area with 3.5 million inhabitants. This Centre specializes in frontotemporal dementia, heredity in dementia, culturally-adapted dementia diagnosis and identifying risk factors for dementia. The main types of research produced by this Centre include neuroimaging databases, intercultural dementia diagnostics and care and diagnostic criteria of familial frontotemporal lobar degeneration. The team at Centre A is bolstered by the collaborative efforts of the departments of Neurology, Neuroscience, Radiology and Nuclear Medicine and Epidemiology
*Centre B* Centre B was established in 2003 and serves a catchment area with 1.5 million inhabitants, and specializes in dementia risk and prevention, biomarkers, diagnostics and disease mechanisms, psychosocial interventions and eHealth. The main type of research produced include a biobank for dementia progression analysis, Living Lab for innovative care environments, artificial intelligence (AI)-based tool for dementia detection and risk reduction and plasma biomarker development for secondary prevention in at-risk individuals​. Centre B involves the departments of Psychiatry and Neuropsychology, Neurology, Radiology and Nuclear Medicine, Epidemiology and Health Services Research to advance dementia care and research
*Centre C* Centre C was established in 2000 and serves a catchment area with 2.5 million inhabitants. Centre C specializes in molecular diagnostics, risk and protective factors, intervention and prevention, early diagnosis and prognosis and neuroimaging to advance understanding, early detection and treatment of Alzheimer’s disease and other dementias​. Centre C involves the departments of Neurology, Psychiatry, Radiology and Nuclear Medicine, Clinical Chemistry, Neuropsychology, Public Health and Genetics, collaborating on dementia research and patient care to enhance diagnosis, treatment and prevention strategies​
*Centre D* Centre D was established in 2019 and serves a catchment area with 1.7 million inhabitants. This Centre focuses on investigates genetic and molecular markers of brain aging and neurodegenerative diseases, a large scale multigenerational cohort study examining health behaviours over the life course to reduce dementia risk, and the TAP-dementia project, a national collaboration aimed at improving dementia diagnosis and incorporating patient feedback​. Centre D involves the departments of Elderly Medicine, Neurology, Neuropsychology, Psychiatry and Radiology
*Centre E* Centre E was established in 2010 and serves a catchment area with 2.1 million inhabitants. This Centre focuses on enhancing long-term dementia care, utilizing AI for better diagnostics, developing innovative imaging technologies and advancing clinical research on Alzheimer’s mechanisms and therapies. Centre E involves the departments of Geriatric Medicine, Neurology, Medical Imaging and Primary and Community Care in its research on dementia care, AI diagnostics, advanced imaging and clinical interventions

### Strategies facilitating knowledge creation

Three main strategies were used by the Alzheimer Centres to facilitate the creation of feasible research knowledge products and accelerate the societal use of research knowledge. First, respondents from all five Centres noted that research funders’ clear guidelines and requirements facilitated implementation planning in the knowledge creation stage, such as by mandating the submission of a dissemination and implementation plan in grant applications. Additionally, funding agencies offered varying research impact models. This guided researchers in developing a “theory of change” to explicate the process toward achieving research impact, beginning with knowledge product development.

“They’re [funders] really working on forming this learning communities, and involving all stakeholders who are also now working on an impact plan. So now we’re really thinking more small in each work pack to also overall for [the consortium] using the theory of change methods from Alzheimer Nederlands, try to see on different levels, ‘Who are you targeting? What are you influencing? What are your bigger goals?’ And really make more visual image plan for impact”. (Respondent 10).

Second, respondents collaborate with diverse cross-sector partners, including government agencies, private organizations and third-sector collaborative partners, to co-create research agendas and knowledge products. For example, Alzheimer Centre researchers received insights into the societal demand for research from patients with lived experiences and healthcare professionals through connections, formed via UMC memory clinics and external events. These events, such as the Dementia Dialogues and Alzheimer Café, focused on dementia knowledge sharing. Research demand was also derived from practice-settings. This includes government officials at the municipality level, national associations (for example, Dutch federation for psychology) and steering committees of regional elderly care networks, consisting of nursing home teams and implementation practitioners.

“What I really learned during this project also is that it’s important to, like in the earlier stages of development, already have the end users involved also. So we always had this neurologist on the team who sees the patients and sees, like the real cases, as they are being discussed at the multidisciplinary meetings and that helps a lot to get this really clinically feasible [diagnostic] tool”. (Respondent 17).

Last, respondents reported actively investing in research system infrastructures that strategically reduce knowledge creation fragmentation (for example, research lifecycle discontinuation), and leverage knowledge beyond project silos. Continuity was achieved by employing research systems interventions, such as long-term public–private (consortium) research projects and learning communities. In addition, Alzheimer Centres A, D and E each hired an Alzheimer Centre coordinator to facilitate the continuation of research projects, through securing subsequent rounds of funding. The coordinator also helped connect interdisciplinary research groups to reduce departmental knowledge silos. Alzheimer Centre A, B and C also emphasized the significance of leveraging formalized public–private partnerships (through research consortia) to reduce knowledge creation fragmentation across sectors.

“We work together a lot because, for example, [name] is the coordinator for the Alzheimer Centre, but also she joins the regional dementia platform. So there are links between the research and the care. For the local GPs, for example, dementia is not an interesting group, but the vulnerable elderly is an interesting group for them, and dementia is a part of this group. So when you make a bit broader, then you have more effect what you’re doing”. (Respondent 25).

“In the new consortia, we’re looking more into co-designing interventions with companies to be part of the application from the start and then also have to contribute in cash or in kind because it makes them more a part of this application. (…) You really have to collaborate with the industry because people are starting to see that only academia driven interventions are usually not the ones that are still used in practice in the long run”. (Respondent 11).

**Table 3:** Strategies identified to facilitate knowledge creation

Broad strategy	Main activities mentioned by respondents from each Alzheimer Centre				
	Centre A	Centre B	Centre C	Centre D	Centre E
(Research funders) provide clear guidelines and requirements for facilitating research impact	Grant applications require dissemination	Funder guides research theory impact roadmap	Results intended for commercial use	Funder required societal impact	Funders required societal impact
(Researchers) co-create research agendas and research knowledge products with external cross-sector partners	Research targets local under-represented needs	Industry partners co-design products	Products tested validated with partners	External partners request tools	Elderly care networks inform researchers
Invest in research system infrastructures that strategically reduce knowledge creation fragmentation and leverage knowledge beyond individual projects	Coordinator reduced research fragmentation	Structural solutions reduce fragmentation	Consortia structure facilitated continuity	Coordinator tracked recruitment efficiency	Coordinator monitors projects and provides guidance

### Strategies facilitating research knowledge product adaptation

Three main strategies were used by Alzheimer Centres to adapt research knowledge products to fit the determinants found in the implementation setting and research ecosystem. First, respondents leveraged resources from the Alzheimer Centres, such as the research, education and healthcare infrastructure, to access patients and participants. This infrastructure can support and inform the adaption of research knowledge to feasible products. For instance, all Alzheimer Centres had access to memory clinics within the UMC, and client panels consisting of people with dementia and their informal caregivers. The proximity to patient groups allowed researchers to seek immediate feedback and adaptation support from the relevant end-users (for example, patient groups and clinicians) to develop culturally appropriate research knowledge products and equitable implementation plans. Alzheimer Centre B, C, D and E also reported training support for researchers, such as for project management, science communication and lobbying and advocacy.

“We have a list of patients who consent to be asked for these things so we can call them, for example. But often of course we set up a specific task and a specific client panel for the project. So in our latest project where investigated feasibility of implementing digital tools from five memory clinics. We asked patients in five memory clinics to comment on the tool. (…) So we included 15 clinicians [including geriatricians] and 40 patients and their significant others”. (Respondent 13).

Secondly, all Alzheimer Centres used wider ecosystem infrastructure and resources from regional networks to adapt knowledge product delivery. For example, respondents adapted the research knowledge product into multiple language options and created simplified, multimodal (for example, print, website, application) versions, closely based on the original intervention components, to increase implementation feasibility and remove end-users’ barriers. Alzheimer Centre A, D and E actively obtained feedback and insights from participants of external networks (for example, regional elderly care networks) to advise the cultural adaptation of research knowledge product components and select implementation strategies that fit the contextual determinants.

“Alzheimer Nederland is a partner in this consortium and Vilans and other partners that really try to translate the research to the public. So we in our junior training programme, there are afternoons that we visit, Alzheimer Nederland or Vilans. I think part of these afternoons was how to involve the public in research design. But also if you have results, how do you try to make the impact that you are aiming for and how to spread the knowledge?” (Respondent 19).

Lastly, respondents utilized the expertise and resources of cross-sector external partners to support knowledge product adaptation. For instance, national knowledge institutes (for example, Vilans, Pharos) acted as implementation support practitioners and knowledge brokers in the dementia research ecosystem. These organizations have trained researchers to use theory of change models to plan research utilization and pragmatically adapt the research knowledge product to fit the infrastructure of the wider implementation ecosystem. Further, funding agencies (for example, ZonMw, Alzheimer Nederland) have also provided technical support to researchers to adapt and communicate research knowledge with cross-sector stakeholders, such as by translating research findings into practical advice to influence policy reform and decision-making.

“You know the way to organize in the Netherlands is the government provides increasingly little direct funding to the university. So there’s still some, but much of the research funding goes through ZonMw [national health research funding agency] and NWO [national research funding agency]. And of course, within these distributing organizations, people focus more on translating the science to policy advice. So that’s their job in particular to try to obtain the results from researchers. Yeah, and they formulate advice to government policy”. (Respondent 2).

**Table 4:** Strategies identified to facilitate knowledge adaptation

Broad strategy	Main activities mentioned by respondents from each Alzheimer Centre				
	Centre A	Centre B	Centre C	Centre D	Centre E
Leverage Alzheimer Centre resources and infrastructures to adapt knowledge products to users	Memory clinics provide access	Adaptation through end-user feedback	Adaptation through events’ feedback	Adaptation through panels' feedback	Specialized department for implementation science
Leverage research system infrastructure and resources to adapt knowledge product delivery and enhance widespread accessibility and usability for end-users	Use regional networks	Industry partner creates software	Develop clinical guidelines for fidelity	Create feasible public products	Adapt findings to new audience
Utilize expertise and resources of external partners to support researchers in facilitating knowledge product adaptation	Understand funding agencies role	Engage industry partners; PhD secondment	National knowledge institute partner	Junior researcher training site visits	Support from knowledge institutes

### Strategies facilitating research knowledge product dissemination

Overall, five main strategies were used by Alzheimer Centres to disseminate research findings and research knowledge products to targeted end-users and relevant stakeholders. First, respondents from each Alzheimer Centre actively sought out, established and fostered cross-sector partnerships between academic institutions, government agencies, private sector (industry) and third sector intermediaries (for example, nonprofit organizations, charities) to overcome traditional knowledge silos. All Alzheimer Centres actively shared research findings through intermediary organization channels, such as national knowledge institutes (for example, Pharos and Vilans), and utilized technical support and science communication training from these organizations. Alzheimer Centres A, C, D and E have received accreditation from health associations, such as the Dutch Federation for Neurologists, to conduct training courses for healthcare professionals. This accreditation facilitates participation from healthcare professionals by offering continuing education credits. Alzheimer Centre C distinguished itself through fundraising activities, merchandise sales and coordination of charity events to disseminate research updates and solicit support from individuals and industry donors.

“We need to make sure that we then send it also to all the funders. So make sure that Alzheimer Nederland has seen it, or ZonMw. (…) Often, for example, funders might say ‘oh, this is a really nice project. We’re so happy to do it together with you, very willing to write something for your website or an interview’”. (Respondent 13).

“And then the wish was to have more in-service training, with credits or points. For the symposium, I also arranged to pick up points as well. Then maybe that helped with the [clinician] attendance numbers. But of course they deserve it. They learn a lot during those days. So there was a wish for more in-service training. So we did a pilot this year and it was very well received”. (Respondent 18).

Second, respondents from Alzheimer Centres A, B and E shared the importance of establishing direct connections with government agencies responsible for updating best practice. A range of activities were reported across each Alzheimer Centre. Main research findings and implications were presented through a ministry report to inform policy. Existing connections and partnerships to were utilized optimize dissemination efforts. Researcher acted as advisors to support the National Dementia Strategy, and they communicated directly with influential political figures through research consortia events. Researchers also engaged funding agencies to act as knowledge brokers with government agencies.

“I’m also in the Advisory Board of the National Dementia Strategy of the Ministry of Health, Welfare and Sport. So every three months we come together, also with the Minister, to talk about dementia and what are gaps, what we have to do. And so I think we have nice channels also to send our message”. (Respondent 7).

Third, multimodal formats of education were used by Alzheimer Centres to disseminate research knowledge findings across specialized and non-specialized audiences. For instance, common activities of research knowledge transfer included conducting virtual webinars and training workshops for healthcare professionals through YouTube. Knowledge was also shared through the Alzheimer Café events and across regional professional networks. These channels foster dissemination beyond the professional networks of the research teams.

“We have a strong connection there and we also have warm links with other Alzheimer cafes so some of them ask us every year for specific sessions to be presented there and also present an overview of new insights in Alzheimer’s disease or new insights in dementia”. (Respondent 8).

“We have had a webinar about this topic last week, explaining more about how to do cross cultural dementia diagnostics as a neuropsychologist, and that was also within our strategy to reach as many healthcare professionals as possible at once. So everybody can watch it. And so that’s step one of the plan: reach as many people as possible”. (Respondent 1).

Fourth, the Alzheimer Centres used social and professional networking applications and commercial marketing strategies to disseminate research knowledge. Each Alzheimer Centre leveraged connections with the communications team from the UMC to share research findings through their social media accounts, marketing channels and official website and newsletter. Alzheimer Centres A, B and C strategically used social and professional networking applications, by creating dedicated webpages on LinkedIn (LinkedIn Corp) and Twitter (X Corp). Alzheimer Centres A and C monitored dissemination outcomes through web and social media analytics tools to incorporate engagement metrics, including total reach and post impressions, but did not use the data to select or tailor dissemination strategies.

“I help with the communication activities and make sure that after every publication the students write a blog, and they share it online and they make an overview of one PowerPoint slide of what the study was about and what are the results. So we have the collection of all those slides, of all the results of the studies, and we use it in presentation”. (Respondent 16).

“You have your different channels; we have our own social media channels. We have newsletters, we do a lot of public lectures. We have Alzheimer’s cafes that a lot of people are involved in the region. So dissemination is something that we really love”. (Respondent 7).

Lastly, formal research support structures from the Alzheimer Centre and external partners, including formal public–private collaborations and regional care networks, were used to facilitate research dissemination. Alzheimer Centres D and E each hired a coordinator to manage and facilitate all dissemination activities, including sharing new research findings via social media and internal and public newsletters, creating a formal communication strategy and actively maintaining relationships with partners (for example, steering committees of regional networks, client panels). Alzheimer Centre C provided more formalized internal structures to disseminate research knowledge, such as science communication training and meetings for researchers to share about their ongoing research projects and standardized templates used for tracking and reporting research outputs for annual reports (for example, consortia research output tracker) and knowledge sharing through social networks (for example, LinkedIn post template).

“For example, in the [consortium], we have outlined all the different target groups that we’re interested in because we also have an aim in that consortium to reach the healthcare professionals. So there we did a kind of mapping of who are the health care professionals that we want to target, and how can we reach them and in what way are we going to reach them?” (Respondent 8).

**Table 5:** Strategies identified to facilitate knowledge dissemination

Broad strategy	Main activities mentioned by respondents from each Alzheimer Centre				
	Centre A	Centre B	Centre C	Centre D	Centre E
Actively seek out, establish and foster cross-sector partnerships between academic institutions, government agencies, private sector (industry) and third sector (intermediaries) to overcome traditional knowledge silos	Funders as knowledge brokers	Industry partner app creation	Keep funders intermediaries updated	Intermediary organizations support dissemination	Intermediary organizations support brokering
Establish direct connections with government agencies responsible for updating best practice guidelines to influence health policy	Identify and advise federations/ committees	Researchers on national board	n/a	n/a	National group for dementia care standard disseminates research
Using multimodal formats of education to disseminate research knowledge findings across diverse (specialized and non-specialized) audiences	Virtual webinars for professionals	Integrate research into courses	Annual lectures to stakeholders	University network collaborates for participation	Knowledge sharing through networks
Use media and market communication strategies to disseminate research knowledge	Disseminate through UMC channels	Dissemination through social media	Alzheimer’s Centre targeted dissemination	Dissemination through UMC channels	Dissemination through UMC channels
Leverage research support structures from the Alzheimer Centre and external partners to facilitate dissemination	Communications manager strategic dissemination	Dissemination through partnerships collaborations	Engage professionals with seminars	Tailor dissemination to specific (niche) audiences	Leverage established networks and partnerships

### Strategies facilitating research knowledge product implementation

Four main strategies to implement, scale-up and sustain research knowledge products across various implementation settings were reported by respondents. First, respondents from each Alzheimer Centre reported the importance of nurturing cross-sector partnerships with government, industry vendors, charities, patient representative groups, funders and collaborative networks. Respondents also reported the value of facilitating such partnerships to adopt and sustain research knowledge products within existing the infrastructure and workflow of industry and third-sector partners. For example, these partnering organizations purchased and implemented the research knowledge product, such as a training module for nurses. The organizations embedded the training module to their website to continue providing education to end-users. Alzheimer Centres B, D and E emphasized the importance of maintaining partnerships with industry to foster trust, ensure continuous communication and leverage respective resources and expertise for scaling collaboration. Sustained partnerships streamline future research knowledge product implementation and reduce resource waste associated with initiating new collaborations.

“And then we also try to make educational materials for healthcare professionals on this topic We just made them and now available also freely available via Alzheimer Netherlands. We’re working on educational models for healthcare professionals on dementia risk reduction to educate them”. (Respondent 7).

Second, respondents from all Alzheimer Centres performed various activities to scale-up research knowledge products implementation as routine practices within existing organizations. Alzheimer Centre A, C and E implemented new research knowledge products (for example, diagnostic tools) directly into the memory clinics and peripheral clinics within the catchment area, with less resistance since these products were co-created with clinical staff members. Alzheimer Centre B, D and E implemented and scaled-up research knowledge products for use in non-clinical settings, such as by adapting a diagnostic approach suitable for implementation in nursing homes. Similarly, strategies were mentioned for implementation across societal systems (for example, education, welfare, health, environment). For example, a health educational module that promotes understanding and inclusivity of people with dementia fit the pillar of an education curriculum that promoted inclusive citizenship. This cross-system implementation demonstrated how strategically aligned, cross-systems collaboration can help to scale research knowledge products implementation beyond system silos to increase research impact to diverse end-user groups. Alzheimer Centre B, D and E sustained research knowledge products within organizations by providing iterative support to a local champion who employed “train the trainer” strategies to facilitate scale-up within implementing organizations.

“We’re looking always a bit for ways to have an entrance with schools because they’re so busy and often very hesitant. So you have this course about citizenship. It’s obligated for primary schools to teach the children to become good citizens. So there’s a project that kind of fits in like because it’s good citizenship to learn about dementia and to do this”. (Respondent 10).

Third, respondents from Alzheimer Centres B, C, D and E reportedly sought out sustainable (alternative) financing from diverse channels to implement and sustain research knowledge products. Alzheimer Centres B and D actively sought additional funding instruments and opportunities to support implementation and sustainment from both public (for example, government funders, municipality subsidies) and private (for example private foundations) funders. Activities from Alzheimer Centres C and E were partially funded by the revenue obtained through licensing fees and product sales, paid by adopting organizations and end-users. Respondents from Alzheimer Centre B and E attempted to have new research knowledge products covered by health insurance reimbursement channels, which required the products to be (cost-) effective and produce positive health outcomes. However, the precise requirements and process to qualify a new product for reimbursement through health insurers were unclear to respondents. Only respondents from Alzheimer Centre B mentioned reimbursement mechanisms from alternative (non-academic) funding sources, such as the *Stimuleringsregeling E-Health Thuis (SET)*, a government-funded initiative that supports the scale-up of eHealth technologies that facilitate home-based care.

“We also, for example, have funding from SET. And so we have also these pilots in the region, but that’s in [city], where we work together with, for example, case managers and care organizations also to implement it in those regional pilots”. (Respondent 8).

In that line, there are also opportunities to embed the research knowledge product within existing health purchasing policies, such as the sustainable coalition initiative (via health insurer).

“[Health insurer] said that they wanted to include this as a priority area in the strategy of ‘van thuis uit’. It’s care concept in the sustainable coalition of [health insurers]. So they want to fund the intervention”. (Respondent 8).

Lastly, respondents from Alzheimer Centre A, B and C reported the use of transdisciplinary knowledge valorization strategies to move research products to real-world practice settings. Alzheimer Centre A, B and C reported that research knowledge was implemented and scaled-up using commercialization practices (for example, structured processes of production, distribution, marketing and sales). Knowledge of legal and regulatory requirements, such as obtaining CE marking and ensuring GDPR compliance for eHealth products, was also beneficial to structure implementation planning. Technology transfer offices at the central university supported the Alzheimer Centres with developing structured business plans and formal contracts that facilitate collaboration with private sector partners. These offices helped to manage the intellectual property rights and legal ownership of the research, and to remain up-to-date on the latest regulatory guidelines throughout the product development process.

“Another part is the valorization that we also worked on and that was dissemination for commercial studies. And so we also had that in mind, in commercial studies, we want to use this as an outcome measure, that would be possible, but they would need to pay a license fee for using the instrument. And using the scoring algorithm, et cetera.(…) we started out early with thinking about implementation. This could be a model in which we earn some money to sustain the academic development and the clinical implementation”. (Respondent 12).

“We’re also speaking to people of the Technology Transfer Office to see, once we have this model, hopefully in a year or two, what steps do we need to do either right beforehand or afterward, to get the CE marking for instance, to be able to bring to a clinical setting and to use it by other healthcare providers”. (Respondent 2).

**Table 6:** Strategies identified to facilitate knowledge implementation

Broad strategy	Main activities mentioned by respondents from each Alzheimer Centre				
	Centre A	Centre B	Centre C	Centre D	Centre E
Nurture cross-sector partnerships with government, industry vendors, charities, patient representative groups, funders and collaborative networks	Project-based implementation pathways infrastructure	Embed training module website	Validate develop algorithm partner	Develop intervention with government agency	Use established networks
Seek opportunities to support the scale-up of an intervention as a regular service	Implement research into clinics	Fit intervention into curriculum	Memory clinics implement instruments	Change modality reduce costs	Sustain products through continued interprofessional relationships
Seek sustainable financing mechanisms from diverse channels	n/a	Seek alternative funding agencies	Commercialization of algorithm fees	Government funding for nursing homes	Association works on financing
Using transdisciplinary knowledge valorization strategies to move research products to real-world practice settings	Technology transfer office supports (business plan, regulations)	Technology transfer office supports (business plan, regulations)	Stakeholders support projects workshops	n/a	n/a

## Discussion

The results identified a range of real-world strategies that promote dementia research utilization. Successful use of these strategies required each Alzheimer Centre to iteratively engage diverse stakeholders, including individuals with lived experiences, caregivers and practitioners, in co-producing both the research knowledge products and the dissemination and implementation processes. The involvement of multiple stakeholders in research co-production aligns with the principles of integrated knowledge translation (IKT), which aim to develop research directions, through engaged scholarship between researchers and knowledge end-users in practice [11]. This is often facilitated through community-based participatory research and knowledge linkage and exchange [18]. Real-world opportunities and challenges for using an IKT approach to research co-production and utilization were clarified through the perspective of dementia researchers. Results confirmed the real value of engaging cross-sectoral stakeholders and end-users to improve research utilization outcomes, while also highlighting the need for new researcher competencies, such as effectively communicating and facilitating collaborations across multidisciplinary teams [40].

Further, this study found that several research funders mandated the use of IKT, requiring research teams to engage end-users and practice agencies in the co-creation of knowledge products and the implementation plan. However, as critiqued by Holmes and Jones [41], the requirements and criteria set by research funders, guiding the nature and strength of co-creation in funded projects, were often loosely defined. The impact of funder activities that promote co-creation and implementation remains unclear. A separate study on dissemination and implementation activities of international research funders revealed that monitoring and measuring research impact was also a prevalent challenge [42]. Further investment is needed to understand how research impact is monitored and evaluated by various funding agencies across diverse research ecosystems.

The choice of research utilization strategies may also be explained by path dependence theory, which implies that strategies are selected on the basis of each Alzheimer Centre’s development trajectory, past decisions, organizational heritage and team competencies [43]. As political and societal forces cause evolution and revolution within the external research ecosystem, Alzheimer Centres may be vulnerable to risks from path dependency, including poor responsiveness to environmental changes, such as disruptive challenges in partnerships and networks or changes in policy [6, 43]. Risks can be mitigated by enhancing team resilience and responsivity. This can be achieved by strengthening researchers’ competencies at each stage of the research continuum through didactic activities, mentorship and expert consultation, knowledge sharing and specialized financing instruments [44, 45]. At an organizational level, Alzheimer Centres may consider structuring annual researcher performance appraisals to include societal research impact in the assessment criteria. Using impact narrative case studies can highlight the societal value of research, as recommended by the national Dutch Strategy Evaluation Protocol 2021–2027 [46]. The practices of other actors within the wider dementia research ecosystem may also evolve to incentivize and support research utilization scaling, such as funding agencies developing dissemination- and implementation-focused financing instruments [42].

The selection of research dissemination and implementation strategies may also vary on the basis of the typology of research products. Respective positions of research products can be mapped across the translational science pipeline: T1 (conducting basic research), T2 (effectiveness in human clinical trials), T3 (implementation of clinically effective products) and T4 (conducting real-world outcome evaluations) [23]. The typology of research products developed in each Alzheimer Centre is largely influenced by the Centre’s research priority areas. For instance, Alzheimer Centre C focused mainly on conducting fundamental research (T1-T2), including biomarker discovery and (pre-) clinical trials, whereas Alzheimer Centre E focused mainly on conducting applied health research (T3-T4), including the implementation of clinically effective non-pharmacological programmes. Respondents from Alzheimer Centre C commonly reported the importance of fostering bilateral research and development (R&D) partnerships with pharmaceutical companies that relied on the Centre’s research infrastructure and leveraging the advantages of integrated public–private discovery and development networks [47]. In contrast, respondents from Alzheimer Centre E emphasized the value of developing and utilizing participatory knowledge infrastructure in the dissemination, implementation and sustainment of research products. Knowledge infrastructure included social and professional collaborative networks with third-sector organizations [48].

Accordingly, depending on which stage of the translational science pipeline the research product is positioned, researchers require certain sets of competencies to overcome the unique determinants (that is, barriers) that influence research utilization. By applying implementation science knowledge, the research utilization process can be explicated using impact and process models. Specific research utilization strategies can be systematically selected and tailored to address specific determinants. Pragmatic tools, such as the research impact logic model (Jones and Bice [49]), and context-specific implementation planning instruments (Prausnitz et al. [50]) are needed to systematically guide research teams in implementation planning. This approach explicates the research utilization process to help monitor and evaluate the outcomes of their utilization strategies.

Several notable dissemination and implementation strategies were identified in this study. First, a cross-systems collaboration strategy was successfully used to implement a health education programme, originally set in healthcare organizations, in a school curriculum. Bunger et al. [51] determined similar benefits of aligning and leveraging existing resources across systems to improve research product implementation feasibility, fidelity and sustainment.

Second, the use of alternative funding mechanisms was commonly reported as a strategy to financially sustain non-pharmacological research products, through adapting products to fit the reimbursement criteria of certain government-funded initiatives and health insurance channels. Findings from Van Kessel et al. [52] further validated this result and reported that the pricing and reimbursement of non-pharmacological research products (for example, digital health interventions) in the Netherlands are determined by negotiations between care providers, health insurers, the Dutch Healthcare Authority and the National Health Care Institute. However, no explicit requirements or guidelines are available to guide researchers to design a sustainable financial reimbursement plan [52].

Lastly, valorization strategies were employed to implement the research products, but a series of challenges impede this approach. The “Code of Practice on the management of intellectual assets for knowledge valorization in the European Research Area” emphasized the importance of “valuing all intellectual assets” generated through research and innovation activities [53]. However, current practices in academic entrepreneurship and research product commercialization focus on patenting and distributing licensing rights on intellectual property and creating independent spin-offs and start-ups [54, 55]. Resultantly, academic “intellectual assets” with lower commercial value are not valorized and often remain siloed within traditional academic settings. To mitigate this risk, future research can explore how an open innovation approach can be applied to dementia valorization, such as by establishing formalized living labs with contributions from cross-sector partners [56]. Best practices from this interdisciplinary method can support stakeholders in the research ecosystem to adopt systems-thinking for knowledge management. It can also enable exploring alternative business models (for example, social enterprise) and feasible implementation pathways for non-traditional research products [57]. As emphasized by Marr and Phan [55], the activities performed by university technology transfer offices to facilitate the valorization of products with lower commercial value are enigmatic. Further systematic exploration of strategies used within such support teams is required to explicate the determinants surrounding the valorization of such products and to create a mutually beneficial link between implementation science and research valorization.

This study may have potential research design and data collection limitations. Purposive sampling was used to recruit respondents, which may introduce selection bias and limit the generalizability of the findings to other settings outside the five included Alzheimer Centres. Another limitation is related to our specialized focus on research utilization and implementation science. The implementation science jargon used by the interviewers required frequent clarification for the interviewees. The need to explain specific terms and concepts might have influenced participants’ answers, as they may have provided responses on the basis of their interpretation of the clarified terminology rather than their initial understanding. This challenge may be influenced by the early stage of implementation science in dementia research in the present Dutch context. Data collection was conducted in English, but some language and cultural nuances shared by respondents, who were native Dutch speakers, may not have been adequately captured. Respondents were given the opportunity to further elaborate on ideas in Dutch to mitigate miscommunication risks. Any elaborations shared in Dutch were discussed and interpreted with the native Dutch speakers in the research team. While in-depth insights were gained, the study’s conclusions should be considered within the context of these limitations.

## Conclusions

Results from the Alzheimer Centres suggest that successful research utilization of non-pharmacological dementia research products requires academic health science centres to build research capacity and develop researcher competencies. This facilitates co-creation with end-users, establishing and maintaining collaborations with public and private partners, and facilitating implementation, scale-up and sustainment. Researchers must take initiative to scale their products, integrating them into existing organizations across sectors and navigating systems to secure inclusion in reimbursement schemes. Using the KTA framework from the perspective of researchers revealed the intricacies involved in streamlining research utilization. That may pave the way for future implementation science studies to enhance the monitoring and evaluation of the research utilization processes, delineated between research producers and users, across various contexts. Employing a comprehensive ecosystem approach ensures the broader impact and practical application of research findings in real-world settings.

## Supplementary Information

Additional file 1.

## Data Availability

The datasets generated and/or analysed during the current study are not publicly available to preserve the privacy and integrity of participants, but data may be made available from the corresponding author upon reasonable request.

## Abbreviations

KTA: Knowledge-to-action (framework)
UMC: University medical centre
IKT: Integrated knowledge translation
NWO: Dutch Organization for Scientific Research
ZonMw: ZorgOnderzoek Nederland (Care Research Netherlands)
SET: Stimuleringsregeling E-Health Thuis (Incentive Scheme E-Health At Home)
