# Supplementary material for: Transforming dementia research into practice: a multiple case study of academic research utilization strategies in Dutch Alzheimer Centres
Source: Health Res Policy Syst. 2025 Jan 6;23:3. doi: 10.1186/s12961-024-01266-9 (PMC11702214; doi:10.1186/s12961-024-01266-9)
Supplement: Supplementary file 1 — Additional file 1. [file 12961_2024_1266_MOESM1_ESM.docx]

Additional file 1

**Table 1. Interview guide**

**Table 2. Abductive coding tree**

**Table 3. Consolidated criteria for reporting qualitative studies (COREQ): 32-item checklist**

| **Table 1. Interview guide** | |
| --- | --- |
| General questions | - Can you please tell us about a research project that you’re most proud of? - Was it successful? What made this successful? - Can you provide examples of projects that have had a significant impact on policy, practice, or the community? |
| RQ 1 (What strategies were used by the Alzheimer Centers to facilitate creation and adaptation of research findings into research products?) | - Could you tell me about a research project that has produced knowledge products that are used in a real-world practice setting, such as guidelines, films, brochures? - Can you elaborate on how these products were created? Who was involved? - Did you receive any formal support from structures within the Alzheimer center or informal (uncontracted) support from your own network? - How did you enhance usability and relevance of the knowledge products for end-users? Did you include the practitioners or patients in this process? |
| RQ 2 (What strategies were employed to disseminate research products?) | - When you get some amazing research results, how do you decide where and how to share it with the public? - How do you identify and target different audiences for your knowledge dissemination activities? - Can you tell me about a dissemination plan you’ve made and how you experienced these knowledge sharing activities? - Can you share a bit about the tools or strategies you use to reach a wider audience with your research findings? - What communication channels do you utilize to disseminate research findings? - How do you measure the impact and effectiveness of your knowledge dissemination efforts? |
| RQ 3 (What strategies were used to facilitate the implementation of research products?) | - How do you foster collaboration with researchers, policymakers, and practitioners to facilitate knowledge use? Are there any notable partnerships that have been instrumental in your center's success? - In your previous or ongoing projects, what implementation (and scale-up) activities have been undertaken? Who is responsible for motivating change? - How do you encourage implementation/scale-up initiatives and knowledge sharing? - How do you monitor the progress and effectiveness of your implementation plans? |

| **Table 2. Abductive coding tree** | | | |
| --- | --- | --- | --- |
| **Deductive lens (KTA)** | **Second-order themes** | **First-order concepts (semantic codes)** | **Direct quotes and examples reflecting each concept/theme** |
| Knowledge creation | (Research funders) provide clear guidelines and requirements for facilitating research impact | - Grant applications require a dissemination and implementation component, so knowledge sharing is part of the job | It's something that ZonMw wants as well. I mean, it's also something that society wants more and more. It's more on the foreground, but it's also something that ZonMw explicitly asks for. (Respondent 22)  One of the conditions, that EU [commission] gave us as a project was that we needed to start a company to ensure valorization of the developed tool. (Respondent 13) |
|  |  | - Funders provide a ‘theory of change methods’ and strategically plan an impact roadmap to create (and co-create) usable knowledge products and deliver them to practice settings | They're [funders] really working on this also on this, forming this learning communities, and involving all stakeholders who are also now working on an impact plan. So now we're really thinking smaller in each work pack to also overall for [the consortium] using the theory of change methods from Alzheimer Nederlands, try to see on different levels. Who are you targeting? What are you influencing? What are your bigger goals? And? Yeah, really make more visual image plan for impact. (Respondent 10) |
|  | (Researchers) co-create research agendas and research knowledge products with (external, cross-sector) partners | - Direct contact with healthcare professionals (via UMC memory clinics, associations, steering committees, …) provides direct insight about real-world knowledge and training demand. | What I really learned during this project also is that it's important to, like in the earlier stages of development, already have the end users involved also. So we always had this neurologist on the team who sees the patients and sees, like the real cases, as they are being discussed at the multidisciplinary meetings and that helps a lot to get this really clinically feasible tool (Respondent 17) |
|  |  | - External events (Dementia Dialogues, Alzheimer Café), and consulting local municipalities and third-party agencies (e.g., case managers, Dutch federation for psychology) | We've had contact previously with the Dutch Heart Foundation and that we spoke about the certain topic that we were interested in and people were gauging what they felt like, what was the information that they said they needed, say, for the public education campaigns, and on the basis of that, we try to shape the project a bit further and come up with some results. We have some results now. We will feed them back. Later on when we have the details worked out. So then their input was there in a bit earlier phase to maybe steer and specify the question we had to build it so that it would be helpful to them as well. *(Respondent 2)* |
|  |  | - Elderly care network steering committee and organizational partners inform Alzheimer Center researchers of both the (national and regional) government agenda, and practice setting needs, to co-develop research projects and feasible knowledge products | I think that's all very relevant to communicate with them about what you want to do with certain findings eventually, and whether that's something they want to get into a guideline, to speak to the people in the steering committees of the associations to be able to see if it's something that is of relevance when they update the guidelines. *(Respondent 2)* |
|  |  | - Partners were involved in designing implementation in early stages as part of the research team and a partner was involved in creating the product and licensing the product to interested parties | So via this company, we were able to really valorize and implement the developed tool. And it's a finished company now, so we don't own it or anything, but it was really, really nice project to see that we really were able to bring something to the market. *(Respondent 13)* |
|  |  | - Create knowledge products based on the needs of the local (underrepresented) population served by the Alzheimer Center | We start with what are the needs and wishes of the people with dementia here and the caregivers and the professionals. In this case, the nurses and the managers in the nursing homes, and then we find out what are crucial aspects and components. And then we look what fits best and therefore we could take some parts of the intervention in it. *(Respondent 29)* |
|  | Invest in research system infrastructures that strategically reduce knowledge creation fragmentation and leverage knowledge beyond individual projects | - Employed an Alzheimer Center coordinator to ensure connectivity between inter-departmental research groups and a project manager for research consortia | I think the biggest benefit is that we have these different departments working together. So we all have our own expertise. So what we do is that the older patients will go to the geriatrician to younger ones, go to the neurologist because that's exactly what separates their expertise and they can help each other in consultations when necessary. But I think we get the best of both worlds in that way. *(Respondent 1)* |
|  |  | - External networks, advisory boards, steering committees, and patient client panels are actively involved to guide the Alzheimer Centers research direction | We work together a lot because, for example, [name] is the coordinator for the Alzheimer Center, but also she joins the dementia platform of Network Honderd. So there are links between the research and the care. But the care …what I told you…for the local GP's, for example, dementia is not an interesting group, but the vulnerable elderly is an interesting group for them, and dementia is a part of this group. Yeah. So when you make a bit broader, then you have more effect what you're doing. *(Respondent 25)* |
|  |  | - Consortia research structure reduces knowledge fragmentation (between specializations, such as diagnosis and patient care) and research discontinuation by engaging partners/stakeholders in practice settings | Also in the new consortia, we're also looking more into co-designing interventions with companies that they also have to be part of the application from the start and then also have to contribute in cash or in kind because it makes them more a part of this application. I believe it's also a bit changing now, also ZonMw and all these other organizations are really looking into more of these TKI [Top consortium for Knowledge and Innovation] applications so you really have to collaborate with the industry because people are starting to see that only academia driven interventions are usually not the ones that are still used in practice in the long run. *(Respondent 11)* |
| Knowledge adaptation | Leverage Alzheimer Center resources and infrastructures to adapt knowledge products to users | - Knowledge product adaptation is facilitated through collecting feedback from end-users (e.g., patients, caregivers, clinicians) via client panels, memory clinic staff, and specialized (European) working group. | Of the 20 organisations, there is our client panel and our researchers often … they have a letter to inform people about their research…then we send it to the client panel to ask “hey, would you read this and is this attractive to read and do you understand what I'm saying? Or do you have well advice for me?” So that way we try to bring the client's voice in our research. *(Respondent 20)* |
|  |  |  | We have a list of patients who consent to be asked for these things so we can call them, for example. But often of course we set up a specific task and a specific client panel for project. So in our latest project where investigated feasibility of implementing digital tools from five memory clinic. We asked patients in five memory clinics to comment on the tool. (…) So we included 15 clinicians [including geriatricians] and 40 patients and their significant others. *(Respondent 13)* |
|  |  | - Participants provide cultural insights and recommendations for communicating the knowledge effectively to non-specialists | We can think of this model, but you know before it's actually going to be used. How would people, how would general practitioners, how would how would individuals citizens respond to such a model? They were confronted with it, now we have some sense of what the model would look like. Now we're asking GPs. Now we're asking participants from our study, how would you feel about implementing such a model? *(Respondent 2)* |
|  |  | - Obtain formal and informal training for science communication (e.g., translating research findings, lobbying), from specialized courses and senior peers, respectively | And we as seniors coach them in how to bring across their message and do that in ways that it's understandable for this lay public. It's not that we have a professional course or coach; it's more that the seniors help the juniors in learning this. We give also feedback to them about this. *(Respondent 8)* |
|  | Leverage research system infrastructure and resources to adapt knowledge product delivery and enhance widespread accessibility and usability for end-users | - Promote accessibility, by providing language translations and various modalities (e.g., print, website, application), to support varied users | It’s not easy to develop the software. But it was a spinout company of [UMC]. It's now being taken over by an American company, but back then it was a spin out of the department, together with [spinoff company] and our population based imaging within the study developed these models and tools, and then we started applying them and validating them and seeing how well they worked within the Alzheimer's center and then transporting things into routine care. So I think that's a very nice example of how you can produce some research and then do also do the validation research, including before you start using it. (Respondent 2) |
|  |  |  | We have a whole campaign. This is the whole booklet that we had…that's the campaign and all the leaflets that we had, all the things that we did, giving lectures, we were on television, we were on marketplace. So there is the app that we developed, the pharmacy action set that we transferred, people get a medicine and also the facts of the campaign, and, in the supermarket, we had an inflatable inflammatory brain here in the hospital. (Respondent 7) |
|  |  | - Adapted version of original product was needed to fit the organizational and cultural context. New adaptations across various contexts were made to ensure generalizability, feasibility and wide suitability | There is a danger of not being a true representative of what's going around in a community. And I think that's one of the reasons why [Alzheimer Center] is also looking at different centers now to collaborate more and then to turn around into a different way of working, with different memory clinics, so the smaller memory clinics, and then do these types of examinations, and is it as specific or as accurate as we think it is, our new diagnostic tool, as most of the times it doesn't end up in the large population. That it is not very accurate. So in a very small group of highly specialized or highly selective cases, it's very, very accurate, but it's not turning out to be accurate in a larger community based group. (Respondent 23) |
|  |  | - External network can help them develop a contextually appropriate implementation plan and select strategies | I think that the best option when performing this kind of research collaboration, the best option is to work with centers in your area. Since the contacts can be easier and closer. So if we would have this project, I think I could think of going, let's say, until Delft. Then Rotterdam, then up to Delft, and down until Dordrecht. So you can get a large area and more patient mixed population. This is also important of course, because you must be able to extrapolate your results to a general population. (Respondent 3) |
|  | Utilize expertise and resources of external partners to support researchers in facilitating knowledge product adaptation | - Understanding the role and applying practices of funding agencies and opportunities to facilitate knowledge translation (and communicate with policy makers) | You know the way to organize in the Netherlands is the government provides increasingly little direct funding to the university. So there's still some, but much of the research funding goes through ZonMw and NWO. And of course, within these distributing organizations, that's people who focus more on translating the science to policy advice. So that's their job in particular to try to obtain the results from researchers. Yeah, and they formulate advices to government policy. (Respondent 2) |
|  |  |  | We also made an Advisory Board during the project with ZonMw, for this years and then we met online and we presented things we did and they could give feedback. We also had one person with dementia, who also connected to Alzheimer Nederland. We also had an online chat of about half an hour, an hour, to look to the lesson and things that she would like to see in the lesson. (Respondent 10) |
|  |  | - Receiving support from knowledge implementation specialists to plan for implementation in early project stages; create a theory of change to guide the planning process | Alzheimer Nederland is a partner in this consortium and Vilans and other partners that really try to translate the research to the public. So we in our junior training program, there are afternoons that we visit, Alzheimer Nederland or Vilans. I think part of these afternoons was how to involve the public in research design. But also if you have results, how do you try to make the impact that you are aiming for and how to spread the knowledge? (Respondent 19) |
|  |  |  | And one other partner of the Board consortium is Pharos. I didn't use them yet with this dissemination. But yeah, they can support the researchers within the consortium with information letters (Respondent 19) |
|  |  |  | If I want to know what is going on among patients and relatives, then I ask Alzheimer Nederland, and I cannot find that within our Alzheimer's Center. Yes, and Vilans is more the connections with the healthcare providers, I think one of their tasks is to implement interventions that help healthcare professionals. If I want to know how I would implement an intervention, or how I would study this…I think I can ask them and I will not find that knowledge in our Alzheimer's center. (Respondent 19) |
|  |  |  | We have a collaboration in the project with Vilans and have asked Vilans to help us think of implementation beforehand, so we're using a theory of change to help us guide also the thinking about implementation. So we made ‘effects karten’, effects card, but that's kind of new for us working with such a well described way of thinking about implementation. Also using the tools that have been developed from theory of change. (Respondent 28) |
| Knowledge dissemination | Actively seek out, establish, and foster cross-sector partnerships between academic institutions, government agencies, private sector (industry), and third sector (intermediaries) to overcome traditional knowledge silos | - External agencies provided continuing education credits accreditation through the Alzheimer Center training courses | The NVN, which is the Dutch Federation for Neurologists, which gathers all neurologists in the Netherlands. So we ask, when you when you get accreditation from them, they will also put you on their agenda and that's mostly what people use if they don't follow our channel. So they are not informed through us and they will see it on that agenda and they will see throughout the year. What can I do because they also have to gain these points to keep up with their accreditation status so they will look through the agenda, what's going on, what can I do, what kind of training am I interested in and it will find us there, hopefully. (....) They have to reach a certain amount of hours yearly or every five years. I think there's a reevaluation of their status. And if they don't have enough of these points, they will lose their status as a neurologist. (Respondent 1) |
|  |  |  | And then the wish was to have more nascholingen (continued education). With points. For the symposium, I also arranged to pick up points as well. Then maybe that helped with the with the attendance numbers. But of course they deserve it. They learn a lot during those days. So there was a wish for more nascholingen (continued education). So we did a pilot this year, and it was very well received. (Respondent 18) |
|  |  |  | The impact is really big there we achieve to really make a difference in people's lives, which is of course different for a healthcare professional because they already know a lot about the subject and that's the yearly annual nascholing [continuing education] (Respondent 15) |
|  |  | Scale up and spread the research product through intermediary organizations (knowledge brokers) and utilize support (e.g., science communication training) from these organizations (Alzheimer Nederland, Pharos, Vilans) | We need to make sure that we then send it also to all the funders. So make sure that Alzheimer Nederland has seen it, or ZonMw. (…) Often, for example, funders might say “oh, this is a really nice project. We're so happy to do it together with you, very willing to write something for your website or an interview.” (Respondent 13) |
|  |  | - Alzheimer Center conducted fundraisers by selling merchandise and coordinating charity events (e.g., concerts, dinners) to disseminate research updates and encourage support from donors (individuals and industry) | I mean, we had a lot of young researchers to give a pitch, for example, at the time [Alzheimer Center leader] gave a nice presentation. There's always content at all of our events, even though it might look that it's not very…it might look different, but as always contents involved because that's what our people who visit us, either it is in a charity dinner or a or genootschaps diner [society dinner] whatever, they want to have the contents, they want to know what the update is. They want to know what [Alzheimer Center leaders] are doing. They want to know what the future will bring them. (Respondent 15) |
|  |  |  | There we have, for example, semi annual meetings where all stakeholders are invited so and then there's always a presentation given by a stakeholder, for example, [company] or Alzheimer Nederland or the municipality on how they look upon specific dementia related subjects. I think they're really well at writing and annual report from the Alzheimer Center that spread out wide, also to inform stakeholders. (Respondent 13) |
|  | Establish direct connections with government agencies responsible for updating best practice guidelines to influence health policy | - Present main findings and important takeaways through a ministry report to inform policy | Also our effectiveness and cost effectiveness studies were included in ministerial reports in different countries and also in the Netherlands it was given as a report to the Minister and therefore it's more and more known. (Respondent 29) |
|  |  | - Utilizing existing connections and partnerships to optimize dissemination efforts, such as a role on national advisory board brings real challenges to a policy level and given immediate consideration | I'm also in the Advisory Board of the National Dementia Strategy of the Ministry of Health, Welfare and Sport. So every three months we come together, also with the Minister, to talk about dementia and what are gaps, what we have to do. And so I think we have nice channels also to send our message. (Respondent 7)  I think Alzheimer Nederland really has because they're really involved, so I think also because they’re also in this Advisory Board. I think is something we should now really do together. I think it's actually the next step here that we are thinking is expanding to indeed do this locally to starting nationally. (Respondent 10) |
|  |  | - Communicate with policymakers through consortia engagement, facilitated by funding agencies that work directly with the government | We have had contacts with the group that developed the dementia standard, the national dementia standard, and that group also helps us a bit to disseminate our results. (Respondent 25) |
|  |  |  | The dementia conference that was organized in The Hague last October [2023] and it was organized by policymakers or the Ministry of Health in the Netherlands, but also the World Dementia Council, and I also experienced how lobbying goes. So I think I already have experienced how important it is to know people and policymakers, and how to get things done. (Respondent 19) |
|  |  | - Identify and advise steering committees of associations (of neurologists and geriatricians) responsible for refining and updating (clinical) best practice guidelines based on new evidence | We proposed our idea and they also agree with us that it was very important. (…) We presented that also on the yearly conference of them. So quite large audience to know that, and yeah, after that, we also heard that new memory clinics, or also other clinics, they said, “well, we changed our protocol into that guideline” and I think that is what you want. You want that when people are thinking about new aspects in their test battery that they then think “well, there is a guideline so we commit to the guideline”, yeah. (Respondent 9) |
|  | Using multi-modal formats of education to disseminate research knowledge findings across diverse (specialized and non-specialized) audiences | - Conducting virtual webinars and sharing via YouTube for all professionals to enhance reach | It is really a postgraduate course and yeah, the same is offered in other countries in Europe, so in England, in France and Italy, and in Switzerland and in Germany, we offered this course and trained OT teachers to offer it now. So I did all those courses and trained all those teachers, but now they are doing it themselves. (Respondent 29) |
|  |  | - Developing training workshop (with continuing education credits) for professionals | We have had a webinar about this topic last week, 4 neuropsychologists, 2 hour long webinar. And explaining more about how to do cross cultural dementia diagnostics as a neuropsychologist, and that was also within our strategy to reach as many healthcare professionals as possible at once. So the webinar was free to watch and people could watch it live. And now today it's been uploaded to our YouTube channel and our website. So everybody can watch it. And so that's step one of the plan. Reach as many people as possible. (Respondent 1) |
|  |  | - Using a direct link to Alzheimer Café and (regional) networks to share knowledge further beyond usual (academic and professional) networks | we have a strong connection there and we also have warm links with other Alzheimer cafes so they some of them ask us every year for specific sessions to be presented there and also present an overview of new insights in Alzheimer's disease or new insights in dementia. So we have several connections to Alzheimer's cafes in the region. (Respondent 8) |
|  | Use media and market communication strategies to disseminate research knowledge | - Dissemination of new research insights from Alzheimer Center shared through UMC marketing channels and the Alzheimer Center website | You have your different channels, so we have a close contact. For instance, if we have a nice message with Alzheimer's Netherlands, the brain foundation, we have our own social media channels. We have newsletters, we do a lot of public lectures. We have Alzheimer's cafes that a lot of people are involved in the region. On a national level. So dissemination is something that we really love. (Respondent 7) |
|  |  | - Strategic dissemination facilitated through social media (LinkedIn/Twitter) and professional networks | So we used that [LinkedIn] actually a lot. I've used that for one of my actually the interview that I've just sent you just to get to people from to get people familiar with what I'm doing because I think that's a little bit difficult at times, especially if you work within a big consortium where you have a lot of PhD students that you have to find like proper ways to make yourself seen. And I think that that interview really helped because. I'd use premium function on LinkedIn. (Respondent 4) |
|  |  | - Employ monitoring practices (e.g., social media analytics) to monitor dissemination strategy effectiveness | So we use like, for social media we have this platform, it's called Coosto and there you can organize all the social media pages and you can see how like the amount that you're growing all the time. (Respondent 5) |
|  |  | - Alzheimer Center employed templates for tracking and reporting research outputs (e.g., consortia result tracker) and for social media dissemination (e.g., LinkedIn post template) | I help with the communication activities and make sure that after every publication the students write a blog and they share it online and they make an overview of one PowerPoint slide of what the study was about and what are the results. So we have the collection of all those slides, of all the results of the studies, and we use it in presentation. (Respondent 16) |
|  | Leverage research support structures from the Alzheimer Center and external partners to facilitate dissemination | - Alzheimer Center employed a center manager or communications manager to facilitate dissemination and develop a formal communication strategy | We were involved in several training networks on technology and dementia, and the researchers there really got into intersectional training, for example, doing secondments, internships for three months in the companies, to get a better grasp of how they are working. They also got more training into from companies. For example, in the way they work and iterations in the way they work, but also about business and about creating impact from a business perspective. So I think that's also important in the future workforce. (Respondent 8) |
|  |  | - Dissemination though existing partnerships and formal research collaborations or networks   - Time and familiarity within the field and networks is a determinant of dissemination success   - Strategically mapping of target groups and planning engagement/ dissemination activities | Structural activities are more overarching over the different projects and there we have tried to link up with the specific projects to see what's the best timing for maybe which project and topic to feed into these overarching activities. And then within the projects we have our specific strategies, for example in the [consortium] we have now from the National Dementia program, we have outlined all the different target groups that we're interested in because we also have an aim in that consortium to reach the healthcare professionals. So there we did a kind of mapping of who are the health care professionals that we want to target, and how can we reach them and in what way are we going to reach them? So then it's more systematically set up, the strategy, and that differs per project how we do that. (Respondent 8) |
|  |  | - Alzheimer Center provides formalized science communication training and meetings for researchers to share (internally and externally) about their ongoing research projects (e.g., Lunch and Learn, Dementia Update) | We have a large Alzheimer conference every summer, AAIC, where Alzheimer's Center is always actively present and we really train hard before that that conference. So I think that really helps as well to sharpen the message of what you have to tell. (Respondent 13)  I'm trying to be active on LinkedIn, we have our own page there from the project and I've also been, as time passed on, I’ve been trying to encourage the PhD students that, , if they go to a conference, presents a poster, or do anything that they would post something on LinkedIn or Twitter with the right affiliations. I've been keeping up with, like, a dissemination list that I can present to the funder if that's necessary. Let's see. But we've been having an internal newsletter for our researchers and our partners. (Respondent 14) |
|  |  | - Alzheimer Center employed templates for tracking and reporting research outputs (e.g., consortia result tracker) and for social media dissemination (e.g., LinkedIn post template) | We have certain guidelines when you know that the publication, for example, is upcoming, then you need to fill out the form and send it to the communication person at the Alzheimer's Center, and then prepare a blog and prepare a LinkedIn. (Respondent 13) |
|  |  |  | Yeah, we have we have a form that we need to fill out. (…) And so, for example, when we have a large conference coming up, then we gotta get a format of what we need to write for LinkedIn. And then every researcher has to do the same and ensure that they post on LinkedIn on that day. (Respondent 13) |
| Knowledge implementation | Nurture cross-sector partnerships with government, industry vendors, charities, patient representative groups, funders, and collaborative networks | - Sustainment through investment or purchasing from an established organization - Embedding online training module (knowledge product) into Alzheimer Nederland website/platform to sustain (with occasional updates from research team) | And then we also try to make educational materials for healthcare professionals on this topic. So we are making that, but also Alzheimer Netherlands, we have now updated the keep your brain fit modules, educational models for the general audience. We just made them and now available also freely available via Alzheimer Netherlands. Also we want to make a new project. We're working on educational models for healthcare professionals on dementia risk reduction to educate them. (Respondent 7) |
|  | Seek opportunities to support the scale-up of an intervention as a regular service | - Implementing new research directly into memory clinics, attached to the UMC and GPs and other primary clinics by adapting products to fit the needs of the demographic of the catchment area | So we are here at a really specialized memory clinics, so we also see special cases, for example, and at the GP setting you will have, well, the a-priori risk of having Alzheimer's disease is of course completely different already. So yeah, that will be really a future step I think. A more advanced stage. But for now we are testing it in our own memory clinic. So the specialized setting, which also includes medical doctors who are also used to looking at new tests and results. (Respondent 17)  But we will also test it in [city], and we also include just our own old age clinic there at the geriatric department. Yeah, to just to test in different settings and see how the medical doctors perceive it, but also how well it aligns with all the diagnostic tests that we have in real world setting.‬ (Respondent 17) |
|  |  | - Adapt and fit the educational intervention into existing infrastructure (e.g., public school curriculum or syllabus) to scale impact | We're looking always a bit for ways to have an entrance with schools because they're so busy and often very hesitant. Like “do we have to do? We don't have time for this”. So you have this burgerschap (citizenship). It's obligated for primary schools to teach the children to become good citizens. But this is, in my opinion, quite vague. Like. It's not specific topics or times, it's just that they should give attention to this topic in that curriculum. So there's a project that kind of fits in like because it's good citizenship to learn about dementia and to do this. So we try to mention this with schools like “oh, you also have to do the citizenship lessons and this would be a nice fit”. But yeah, it's not something formal. So that's maybe a way in I see to have it more implemented. (Respondent 10) |
|  |  | - Continued relationship between research team and cross-sector partner allows for easier communication, trust, and growth over time. Each side contributes their own expertise, and each will support the other in their shortcomings to achieve sustainable scale up - Sustainment of intervention within the organization through continuous contact with a local champion or ambassador and scale using train the trainer strategies | The Dutch Alzheimer Society to now make this project from a local project in the South of the Netherlands to a national project all over the Netherlands. Because they [Alzheimer Nederland] have all this network of volunteers, they all give education about dementia. (…) Now it's really integrated in their education program. Someone is a volunteer and wants to give education, eventually they can use our adoption project and also find schools and care homes. (Respondent 10) |
|  |  |  | We are with five of us in 20 organizations. So we can't do that by ourselves. So we also educate people in the organization themselves. So they are the linking pins for us so that we spread also implementation knowledge, not only knowledge of the researchers, those results, but also implementation knowledge. (Respondent 21) |
|  |  |  | We also have the network of 20 nursing homes that are like committed to research and we also offer them more…We have the implementation advisors and they go to the nursing homes. So they have really more knowledge about how to implement and they know all the steps that you need to do within an implementation effort. (Respondent 22) |
|  | Seek sustainable financing mechanisms from diverse channels | - Seeking alternative funding instruments from diverse funding agencies and financing streams (e.g., foundations, local municipalities, smaller grants, government subsidies) to sustain the intervention in real-world settings - Funding from profit generated by commercializing product | Another part is the valorization that we also worked on and that was dissemination for commercial studies. And so we also had that in mind, in commercial studies, we want to use this as an outcome measure, that would be possible, but they would need to pay a license fee for using the instrument. And using the scoring algorithm, et cetera. So that was also from the start. Yeah, what I mentioned, we started out early with thinking about implementation. This was thought of, well, this could be a model in which we earn some money to sustain the academic development and the clinical implementation. (Respondent 12) |
|  |  | - Fitting the intervention into the criteria/description of the alternative funding requirements. Intervention aligns with reimbursement criteria or health laws, eligible for reimbursement, like government-funded eHealth tech for home-based care. (Stimuleringsregeling E-Health Thuis (SET)) - Pilot studies and effectiveness studies demonstrate intervention impact. If aligned with reimbursement criteria or existing laws, insurers may reimburse through current mechanisms. | They want to evaluate it in a certain number of people and measure the impact. They have a kind of impact tool at [insurance company]. And if it's positive, they want to uh, implement it in the whole of the Netherlands. So they start with the region. (Respondent 8)  [Insurance companies] looking at more overarching, so not just staying in the same lane, but also looking at investments, for instance, Social Support Act (WMO) (Respondent 11)  What kind of benefits do they make in the long term care and they are very interested in [intervention], which is something I'm very happy about, and they are willing to start a pilot next year 2024 in the van thuis uit [from home] program. That is really overarching program designed within the same group of [insurance company], and after this pilot, they will look at the impact is big enough to go for sustainable financing in the future. (Respondent 11)  We also, for example, have funding from the SET regeling (Stimuleringsregeling E-Health Thuis (SET)). Ehealth stimulation at home. And so we have also these pilots in the region, but that's in [city], where we work together with, for example, case managers and care organizations also to implement it in those regional pilots. (Respondent 8)  Yeah, we're currently trying to collect these data within the SET application that we're doing together with the case management as well (…) it was a good a point that you raised about decreasing the face to face contact. And that's what we're currently looking at within the SET application with a few case management organizations to see if we can demonstrate this effect because they all say it, but no one has really put it into numbers yet, so that's what we're trying to do now. (Respondent 11) |
|  | Using transdisciplinary knowledge valorization strategies to move research products to real-world practice settings | - Knowledge product (model/tools) obtained through research were applied, translated/adapted to accelerate/enhance real-world usability | We're still working very much on like the scientific part of it, on developing actually the model that might be good enough to validate that. But at the same time, we're also speaking to people of the technology Transfer Office to see, like, you know, once we have this model, hopefully in a year or two, what steps do we need to do either right beforehand or afterward, to get the CE marking for instance, to be able to bring to a clinical setting and to use it by other healthcare providers. We can think of this model, but you know before it's actually going to be used. (Respondent 2)  I think it's important to keep that into account if we're, if we're speaking about valorization because what you often see is that valorization is associated with that. It needs to reach the citizens and it needs to reach the patients and of course, that's for much of the clinical and the applied research we do, that's super relevant. But as it also very much depends on the type of reasons that we that we do and I think we should also be careful to not overwhelm people with knowledge that may or may not be very robust or replicable. (Respondent 2) |
|  |  | - Knowledge valorization office/technology transfer office at the central university supports AC in developing/managing business plans, formal contracts for private sector partners, staying up-to-date on the latest regulatory guidelines (e.g., GDPR, Medical Device Regulation) | there are all competences which are not, which I was not familiar with, and which was not part of my background. So all these regulations in the English language and all steps in business models or steps in between. Then a short and long term and sustainable business plan. And valorization and all thoughts about that. Yeah. You are not familiar as a researcher with that, as a scientist, but that's part of the job when you develop something and you want that other people are using it. So yeah, we learned a lot. (Respondent 9)  Now they [valorization organization] are also involved in our consortium, and they're actually going to give a talk at our annual meeting on this part of the valorization, like on the biotechnology business side of things. (…) Like, how could our projects and the product, let's say, the diagnostic test battery…how could that actually be realized in biotech and business and further on, but that is something that we have to do with them and still think about. (Respondent 14)  I wouldn't argue that we should already like roll out this blood test to just [commercialize] Yeah, but I noticed, for example, that America, that's what they do. You can just request it. I think in Europe, but maybe the Netherlands specifically, we are a bit more hesitant than that because, yeah, we really think that you should always judge such biomarker results together with the full clinical picture and not just run it on everyone (Respondent 17)  For the PREDICT-AD tool, intellectual property was with the company, and that was decided on beforehand. Now, we recently developed a new decision model for within the tool and there we had to rephrase intellectual property paragraph in the agreement. And now we have so much shared intellectual property, so they used the algorithm that we developed with them, hashtag towards the paper where we described our methods. (Respondent 13) |

| **Table 3. Consolidated criteria for reporting qualitative studies (COREQ): 32-item checklist** | | |
| --- | --- | --- |
| **No. Item** | **Guide questions/description** | **Reported on Page #** |
| **Domain 1: Research team and reﬂexivity** |  |  |
| *Personal Characteristics* |  |  |
| 1. Interviewer/facilitator | Which author/s conducted the interview or focus group? | Page 8 |
| 2. Credentials | What were the researcher’s credentials? E.g. PhD, MD | Page 8 |
| 3. Occupation | What was their occupation at the time of the study? | Page 8 |
| 4. Gender | Was the researcher male or female? | (n/a) not relevant |
| 5. Experience and training | What experience or training did the researcher have? | Implicitly reported (see no. 2 and 3) |
| *Relationship with participants* |  |  |
| 6. Relationship established | Was a relationship established prior to study commencement? | **Page 7** |
| 7. Participant knowledge of the interviewer | What did the participants know about the researcher? e.g. personal goals, reasons for doing the research | Page 7 |
| 8. Interviewer characteristics | What characteristics were reported about the inter viewer/facilitator? e.g. Bias, assumptions, reasons and interests in the research topic | Implicitly reported on Page 7 (reasons were disclosed in informed consent form) |

| **Domain 2: study design** |  |  |
| --- | --- | --- |
| *Theoretical framework* |  |  |
| 9. Methodological orientation and Theory | What methodological orientation was stated to underpin the study? e.g. grounded theory, discourse analysis, ethnography, phenomenology, content analysis | Page 8 |
| *Participant selection* |  |  |
| 10. Sampling | How were participants selected? e.g. purposive, convenience, consecutive, snowball | Page 7 |
| 11. Method of approach | How were participants approached? e.g. face-to-face, telephone, mail, email | Page 7/8 |
| 12. Sample size | How many participants were in the study? | Page 9 |
| 13. Non-participation | How many people refused to participate or dropped out? Reasons? | Page 8 |
| *Setting* |  |  |
| 14. Setting of data collection | Where was the data collected? e.g. home, clinic, workplace | Page 8 |
| 15. Presence of non-participants | Was anyone else present besides the participants and researchers? | n/a (not relevant to this research) |
| 16. Description of sample | What are the important characteristics of the sample? e.g. demographic data, date | Page 7, 9 |
| *Data collection* |  |  |
| 17. Interview guide | Were questions, prompts, guides provided by the authors? Was it pilot tested? | Page 8 |
| 18. Repeat interviews | Were repeat interviews carried out? If yes, how many? | Page 8 |
| 19. Audio/visual recording | Did the research use audio or visual recording to collect the data? | Page 8 |
| 20. Field notes | Were ﬁeld notes made during and/or after the interview or focus group? | n/a |
| 21. Duration | What was the duration of the inter views or focus group? | Page 8 |
| 22. Data saturation | Was data saturation discussed? | Page 8 |
| 23. Transcripts returned | Were transcripts returned to participants for comment and/or correction? | Page 8 |
| **Domain 3: analysis and ﬁndings** |  |  |
| *Data analysis* |  |  |
| 24. Number of data coders | How many data coders coded the data? | Page 8 |
| 25. Description of the coding tree | Did authors provide a description of the coding tree? | Page 8,9 |
| 26. Derivation of themes | Were themes identiﬁed in advance or derived from the data? | Page 8 |
| 27. Software | What software, if applicable, was used to manage the data? | n/a |
| 28. Participant checking | Did participants provide feedback on the ﬁndings? | Page 8 (via transcript checking) |
| *Reporting* |  |  |
| 29. Quotations presented | Were participant quotations presented to illustrate the themes/ﬁndings? Was each quotation identiﬁed? e.g. participant number | Page 11 - 21 |
| 30. Data and ﬁndings consistent | Was there consistency between the data presented and the ﬁndings? | Page 21-25 |
| 31. Clarity of major themes | Were major themes clearly presented in the ﬁndings? | Page 11 – 21 |
| 32. Clarity of minor themes | Is there a description of diverse cases or discussion of minor themes? | Page 11 – 21 |
